# Pro-inflammatory mediators released by activated monocytes promote aortic valve fibrocalcific activity

**DOI:** 10.1186/s10020-022-00433-4

**Published:** 2022-01-21

**Authors:** Peijian Zhang, Erlinda The, Zichao Luo, Yufeng Zhai, Qingzhou Yao, Lihua Ao, David A. Fullerton, Dingli Xu, Xianzhong Meng

**Affiliations:** 1 Department of Surgery, University of Colorado Denver, Aurora, CO 80045 USA; 2 Department of Cardiology, Nanfang Hospital, Southern Medical University, Guangzhou, 510515 China

**Keywords:** Aortic valve interstitial cell, Monocytes, Fibrocalcification, Cytokines, Signaling

## Abstract

**Background:**

Calcific aortic valve disease (CAVD) is the most prevalent heart valve disorder in the elderly. Valvular fibrocalcification is a characteristic pathological change. In diseased valves, monocyte accumulation is evident, and aortic valve interstitial cells (AVICs) display greater fibrogenic and osteogenic activities. However, the impact of activated monocytes on valular fibrocalcification remains unclear. We tested the hypothesis that pro-inflammatory mediators from activated monocytes elevate AVIC fibrogenic and osteogenic activities.

**Methods and results:**

Picro-sirius red staining and Alizarin red staining revealed collagen and calcium depositions in cultured human AVICs exposed to conditioned media derived from Pam3CSK4-stimulated monocytes (Pam3 CM). Pam3 CM up-regulated alkaline phosphatase (ALP), an osteogenic biomarker, and extracellular matrix proteins collagen I and matrix metalloproteinase-2 (MMP-2). ELISA analysis identified high levels of RANTES and TNF-α in Pam3 CM. Neutralizing RANTES in the Pam3 CM reduced its effect on collagen I and MMP-2 production in AVICs while neutralizing TNF-α attenuated the effect on AVIC ALP production. In addition, Pam3 CM induced NF-κB and JNK activation. While JNK mediated the effect of Pam3 CM on collagen I and MMP-2 production, NF-κB was critical for the effect of Pam3 CM on ALP production in AVICs.

**Conclusions:**

This study demonstrates that activated monocytes elevate the fibrogenic and osteogenic activities in human AVICs through a paracrine mechanism. TNF-α and RANTES mediate the pro-fibrogenic effect of activated monocytes on AVICs through activation of JNK, and TNF-α also activates NF-κB to elevate AVIC osteogenic activity. The results suggest that infiltrated monocytes elevate AVIC fibrocalcific activity to promote CAVD progression.

## Background

Calcific aortic valve disease (CAVD) is a leading cardiovascular disorder in the elderly (Yutzey et al. 2014; Otto and Prendergast 2014). This disease is a major health care issue since it causes significant morbidity and mortality (Li et al. 2011). CAVD is characterized by chronic fibrosis and calcification in valvular leaflets, and progressive fibrocalcification eventually results in aortic stenosis that requires surgical or transcatheter aortic valve replacement (Otto and Prendergast 2014). While the slow progression of CAVD offers a wide window for pharmacological intervention, pharmacological therapies are currently unavailable for prevention of this disease progression.

Aortic valve interstitial cells (AVICs), the predominant cells in valvular tissue, are crucial for maintaining valvular tissue homeostasis (Mathieu et al. 2015). However, in the pathophysiological conditions, these cells can undergo phenotype transitions into myofibroblasts and osteoblast-like cells and thereby play an important role in valvular fibrocalcification (Li et al. 2011; Mathieu et al. 2015; Liu et al. 2007). Valvular fibrosis and calcification are characteristic of CAVD pathobiology (Miller et al. 2011; Weiss et al. 2013). In this regard, histopathologic examinations of aortic valves from CAVD patients demonstrated that valvular tissue thickens with excesses of fibrotic materials and calcified nodules (Lindman et al. 2016). Understanding the cellular and molecular mechanisms underlying valvular fibrocalcification would enable the exploration of pharmacological interventions for prevention of CAVD progression.

It is known that CAVD is a chronic inflammatory disease (Miller et al. 2011). Toll-like receptors (TLRs) play a role in mediating pro-inflammatory responses in AVICs (Zeng et al. 2012; Zhang et al. 2020). TLR stimulation in human AVICs promotes the production of pro-inflammatory mediators (Zeng et al. 2012). Many studies, including ours, suggest that inflammation promotes aortic valve leaflet fibrosis and calcification (Mathieu et al. 2015; Song et al. 2012; Zeng et al. 2017). In this regard, pro-inflammatory stimuli are found to elevate the fibrogenic and/or osteogenic activity in human AVICs (Zhan et al. 2015).

Monocytes play mechanistic roles in chronic inflammatory diseases (Suhrbier 2019). Several studies have found that monocytes augment vascular inflammation by secreting pro-inflammatory mediators, and monocytes and pro-inflammatory mediators from them promote atherosclerotic progression (Jaipersad et al. 2014). Monocyte infiltration and macrophage accumulation have been observed in aortic valve leaflets from CAVD patients (Zhou et al. 2020). Our recent study found that monocytes activated by TLR2 agonists enhance the inflammatory activity in human AVICs (Zhang et al. 2020). High levels of pro-inflammatory cytokines secreted by monocytes contribute to the pathobiology of chronic inflammatory disorders such as rheumatoid arthritis and atherosclerosis (Suhrbier 2019; Sun et al. 2021). It has been reported that monocytes promote atherosclerotic calcification via paracrine activity (Jaipersad et al. 2014). As pro-inflammatory factors are capable of inducing the fibrogenic and osteogenic responses in AVICs (Cote et al. 2013; Husseini et al. 2014; Chen et al. 2011), it is reasonable to propose that activated monocytes promote valvular fibrocalcification through paracrine modulation of the production of fibrogenic and osteogenic mediators in AVICs.

In this study, we tested the hypothesis that activated monocytes promote the fibrocalcification in human AVICs. The purposes of the present study were to: (1) determine the effect of activated monocytes on AVIC fibrocalcification, (2) identify monocytes factors responsible for augmentation of fibrocalcification in human AVICs, and (3) elucidate the molecular mechanism underlying the effect of factors from activated monocytes.

## Materials and methods

### Chemical and reagents

Antibodies against phosphorylated extracellular-regulated kinase (ERK) 1/2 (9101), total ERK1/2 (9102), phosphorylated p38 mitogen-activated protein kinase (p38) (9211), total p38 (9212), phosphorylated nuclear factor kappa-light-chain-enhancer of activated B cells (NF-κB) (3034), total NF-κB (3033), phosphorylated c-Jun N-terminal kinase (JNK) (9251), total JNK (9252) and matrix metalloproteinase-2 (MMP-2) (40994) were purchased from Cell Signaling, Inc (Beverly, MA). Antibodies against Alkaline Phosphatase (ALP) (ab108337), type IV collagen (collagen IV) (ab6586) and GAPDH (ab9385) were obtained from Abcam (Cambridge, MA). Antibody against bone morphogenetic protein 2 (BMP2) (XP-5111) was obtained from ProSci (Poway, CA). Antibodies against type I collagen (collagen I) (PA2140-2) and matrix metalloproteinase-9 (MMP-9) (PB9668) were obtained from Boster (Pleasanton, CA). Medium 199 (M199) was obtained from Lonza (Walkersville, MD). Roswell Park Memorial Institute medium (RPMI 1640) was obtained from GIBCO Laboratories (Grand Island, N.Y.). Pam3CSK4 was obtained from InvivoGen (San Diego, CA). Tumor necrosis factor-α (TNF-α) neutralizing antibody (MAB 610), regulated on activation, normal T cell expressed and secreted (RANTES) neutralizing antibody (MAB678) and SP600125 were purchased from R&D System (Minneapolis, MN). Bay 11-7082, Alizarin red S, picaro-sirius red (PSR), collagenase and other reagents were obtained from Sigma-Aldrich Chemical Co. (St Louis, MO).

### Isolation and culture of human primary AVICs

Normal aortic valves were collected from explanted hearts of patients having cardiomyopathy and undergoing heart transplantation. Demographic information of patients is presented in Table 1. The valve leaflets were thin and did not have histological abnormality. This study was approved by the University of Colorado Multiple Institution Review Board (IRB Protocol 08-0280). All subjects gave their informed consent for the use of their aortic valves for this study. The investigations were carried out following the rule of the Declaration of Helsinki of 1975, revised in 2013.

**Table 1:** Demographic information of aortic valve donors

Number	Age (years)	Gender	Diagnosis
Donor 1	62	Male	Cardiomyopathy
Donor 2	58	Male	Cardiomyopathy
Donor 3	60	Male	Cardiomyopathy
Donor 4	63	Female	Cardiomyopathy
Donor 5	59	Female	Cardiomyopathy

AVICs were isolated and cultured using a previously described method (Zhang et al. 2020). Briefly, valve leaflets were subjected to sequential digestions with collagenase. A high concentration of collagenase (2.5 mg/ml) was used to remove endothelial cells. Then, the tissue was digested in a solution containing 0.8 mg/ml of collagenase to free the interstitial cells, and AVICs in the solution were harvested by centrifugation. Cells were maintained in M199 growth medium supplemented with 10% fetal bovine serum, penicillin G (100 units/ml), streptomycin (100 mg/ml) and amphotericin B (0.25 µg/ml). Cells of passage 3–6 were used for the experiments when the cells are approximately 80% confluence.

### Culture of THP-1 monocyte

THP-1 monocyte cell line was purchased from American Type Culture Collection (Manassas, Virginia). According to manufacturer’s instruction, THP-1 cells were cultured in T-75 flask with RPMI 1640 growth medium supplemented with 10% fetal bovine serum in an incubator with 5% CO_2_ at 37 °C. The medium was renewed every 2–3 days. Cells were subcultured at a density of 8–10 × 10^5^ cells/ml.

### Treatment of AVICs with conditioned medium

To generate conditioned medium, monocytes were seeded in 12-well plates at a density of 2 × 10^5^ cells/ml and incubated in medium containing 2.5% fetal bovine serum overnight before treatment. Monocytes were treated with Pam3CSK4 at 0.1 μg/ml for 24 h or left untreated. Culture media were collected after treatment. The supernatant from Pam3CSK4-treated monocyte culture is described as Pam3CSK4 conditioned medium (Pam3 CM). The supernatant from untreated monocyte culture is described as control conditioned medium (control CM).

Conditioned medium was stored at − 80 °C before use. For the experiment, a mixture of 0.15 ml conditioned medium and 0.35 ml growth medium [30% (vol/vol) of conditioned medium] was applied to AVICs in 24 well plate.

To neutralize specific cytokine in the Pam3 CM, neutralizing antibodies against TNF-α, RANTES or both at 10 µg/ml were added to Pam3 CM and incubated for 1 h at 37 °C prior to being applied to treat AVICs. Aliquots of the Pam3 CM were treated with non-immune IgG and used as controls.

To determine the roles of JNK and NF-κB, JNK inhibitor (SP600125, 10 μmol/l) and NF-κB inhibitor (Bay11-7082, 10 μmol/l) were added to AVIC culture 30 min before adding Pam3 CM. DMSO (10 μmol/l) was applied to AVICs as a vehicle control.

To inhibit TLR2 in AVICs, specific TLR2 inhibitor CU CPT 22 (10 µM) was added to AVIC culture 1 h prior to subsequent experiment.

### Alizarin red staining

Alizarin red S staining was performed to identify calcium deposits in AVIC culture. AVICs were cultured in 0.35 mL pro-osteogenic medium (growth medium with 10 mmol/l beta-glycerophosphate, 10 nmol/l vitamin D3, and 10 nmol/l dexamethasone) and exposed to CM (0.15 ml) or Pam3CSK4 (0.15 ml, final concentration 0.03 μg/ml) for 10 days. Cells were washed twice with PBS, fixed with 4% paraformaldehyde, and then incubated in 0.2% alizarin red solution (pH 4.2) for 30 min. The excess dye was washed with distilled water. Alizarin red staining was examined and photographed using a Nikon Eclipse TS100 microscope (Tokyo, Japan). For quantitative analysis, each well was incubated with 10% acetic acid at room temperature for 30 min. Eluted Alizarin red was quantitated by spectrophotometry.

### Picro-sirius red staining

PSR staining specifically identifies collagens and is a useful method for the assessment of fibrogenic activity in cultured cells. AVICs were culture in M199 (0.35 ml) and exposed to CM (0.15 ml) or Pam3CSK4 (0.15 ml, final concentration 0.03 μg/ml) for 14 days. Then, AVICs were treated with methanol overnight at − 20 °C. Cells were washed with PBS and incubated in 0.1% PSR for 4 h. Cells were then rinsed with 0.1% acetic acid, air-dried and examined under a microscope. Cells were then treated with 0.1 ml of 0.1 M sodium hydroxide for 2 h at room temperature to elute the color. The optical density of sodium hydroxide solution was determined using a spectrophotometer (BioTek Instruments, Inc., Winusky, VT, USA) at 540 nm. The results are expressed as % of change compared to controls.

### Immunoblotting

Immunoblotting was performed to assess levels of p-ERK1/2, ERK1/2, p-p38, p38, p-NF-κB, NF-κB, p-JNK, JNK, MMP-2, MMP-9, ALP, RUNX2, collagen I and collagen IV in AVIC lysate. AVICs were washed with PBS and lysed with 2 × Laemmli sample buffer (Bio-Rad Laboratories, Inc., Hercules, CA). Samples of cell lysate were separated on 4–20% SDS-PAGE gels and transferred to nitrocellulose membranes. The membranes were blocked with 5% skim milk solution for 1 h at room temperature, incubated with primary antibodies (1:200 to 1:1000 volume dilutions) overnight at 4 °C, and then incubated with secondary antibodies for 2 h at room temperature. Membranes were developed using the enhanced chemiluminescence system. The levels of detected proteins were normalized against GAPDH. Densitometric analysis of protein band was performed using the ImageLab software (Bio-Rad Laboratories, Inc., Hercules, CA).

### ELISA

Our previous multi-plex analysis identified greater levels RANTES and TNF-α in Pam3 CM (Zhang et al. 2020). We applied ELISA kits (R&D Systems, Minneapolis, MN) to determine the levels of TNF-α and RANTES in conditioned medium. Samples of conditioned medium and standards were prepared according to manufacturer’s instructions. Absorbance of standards and samples were determined spectrophotometrically at 450 nm, using a microplate reader (Biotek, Winooski, VT). Results were plotted against the linear portion of the standard curve.

### Statistical analysis

Statistical analyses were performed using Prism Software (GraphPad). Data are presented as mean ± SEM. ANOVA with the post hoc Fisher test was performed to analyze differences between multiple groups, and t-test was applied to compare data between two groups. Nonparametric Mann–Whitney U test was performed to confirm the difference of two group comparison. For multiple group comparisons, nonparametric Kruskal–Wallis test was performed to confirm the differences. Statistical significance was defined as *P* ≤ 0.05.

## Results

### Conditioned medium from activated monocytes promotes collagen and calcium deposition in human AVICs

**Fig. 1:**
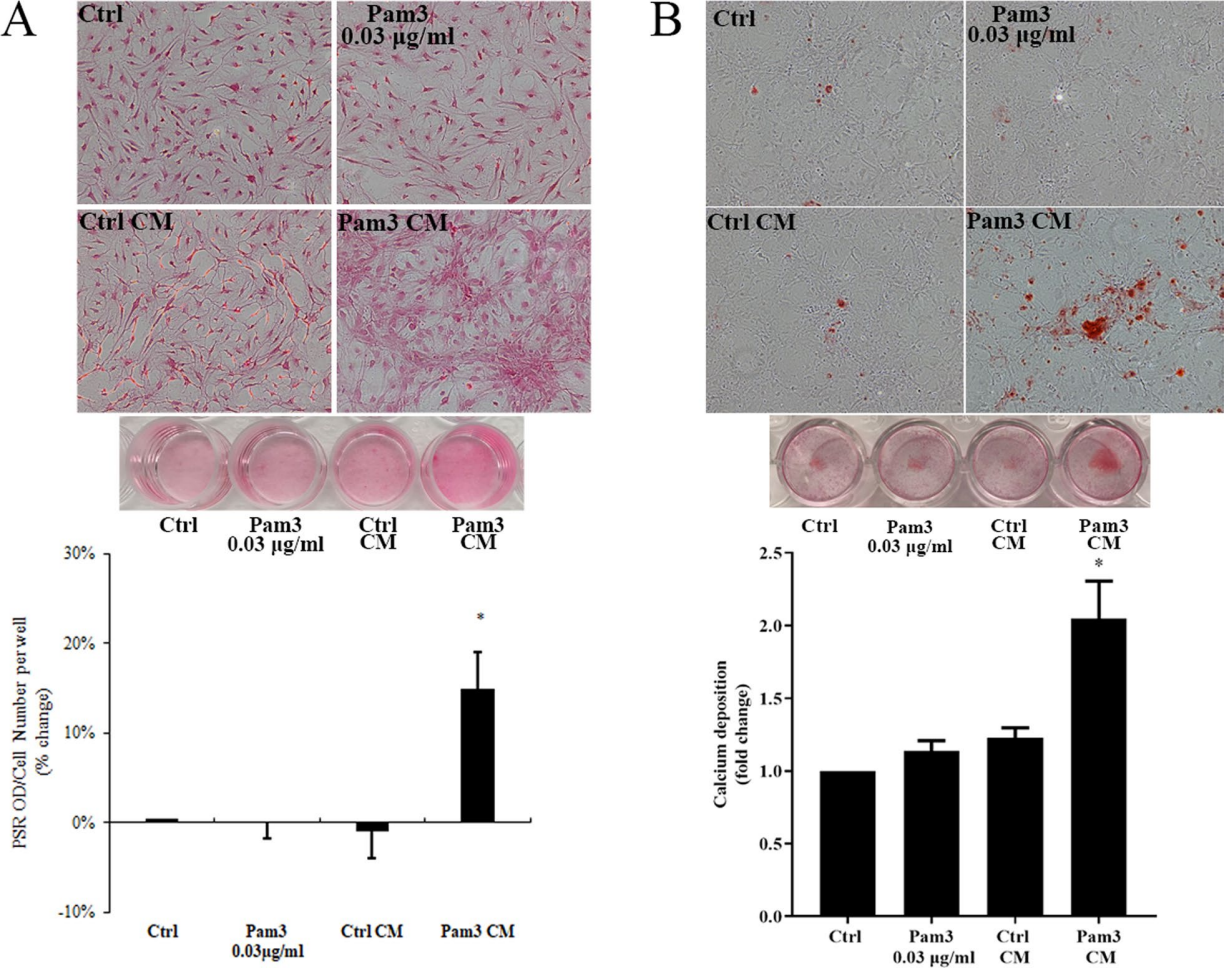
Pam3 CM promotes collagen and calcium deposition in AVICs. **A** AVICs were cultured in the growth medium and treated with Pam3 (0.03 μg/ml), control CM or Pam3 CM for 14 days. Representative images of Picro-Sirius Red staining and spectrophotometric quantitation normalized by cell density show that increased collagen deposition occurred in AVICs exposed to Pam3 CM, not in cells exposed to control CM or a low concentration of Pam3. **B** AVICs were cultured in pro-osteogenic medium and treated with Pam3 (0.03 μg/ml), control CM or Pam3 CM for 10 days. Representative images of Alizarin red S staining and spectrophotometric quantitation show that Pam3 CM, not control CM or a low concentration of Pam3, increased calcium deposition in AVICs. *P < 0.05 vs. untreated control, Pam3 or control CM. All quantitative data are presented as mean ± SEM. Each experiment was performed with cells isolated from 5 different donors

To determine the effect of activated monocytes on AVIC fibrogenic and osteogenic activities, we added conditioned media derived from Pam3CSK4 (0.1 μg/ml)-treated monocytes (Pam3 CM) and untreated monocytes (control CM) to AVIC. Additional AVIC cultures were treated with 0.03 μg/ml Pam3CSK4 (equivalent to Pam3CSK4 level in the AVIC cultures treated with 30% Pam3 CM). Picro-sirius staining revealed that prolonged exposure of human AVICs to Pam3 CM caused greater collagen deposition in comparison to untreated controls (Fig. 1A). In contrast, control CM and the low level of Pam3CSK4 had no effect on collagen deposition. AVICs were also cultured in pro-osteogenic media and treated with Pam3 CM for 10 days. Alizarin red S staining data showed that Pam3 CM greatly increased both the accumulation of calcium deposits and the formation of calcified nodules in AVICs (Fig. 1B). These data indicate that monocytes activated by a TLR2 agonist promote AVIC fibrogenic and osteogenic activities.

### Conditioned medium from activated monocytes enhances the production of fibrogenic and osteogenic factors in AVICs

**Fig. 2:**
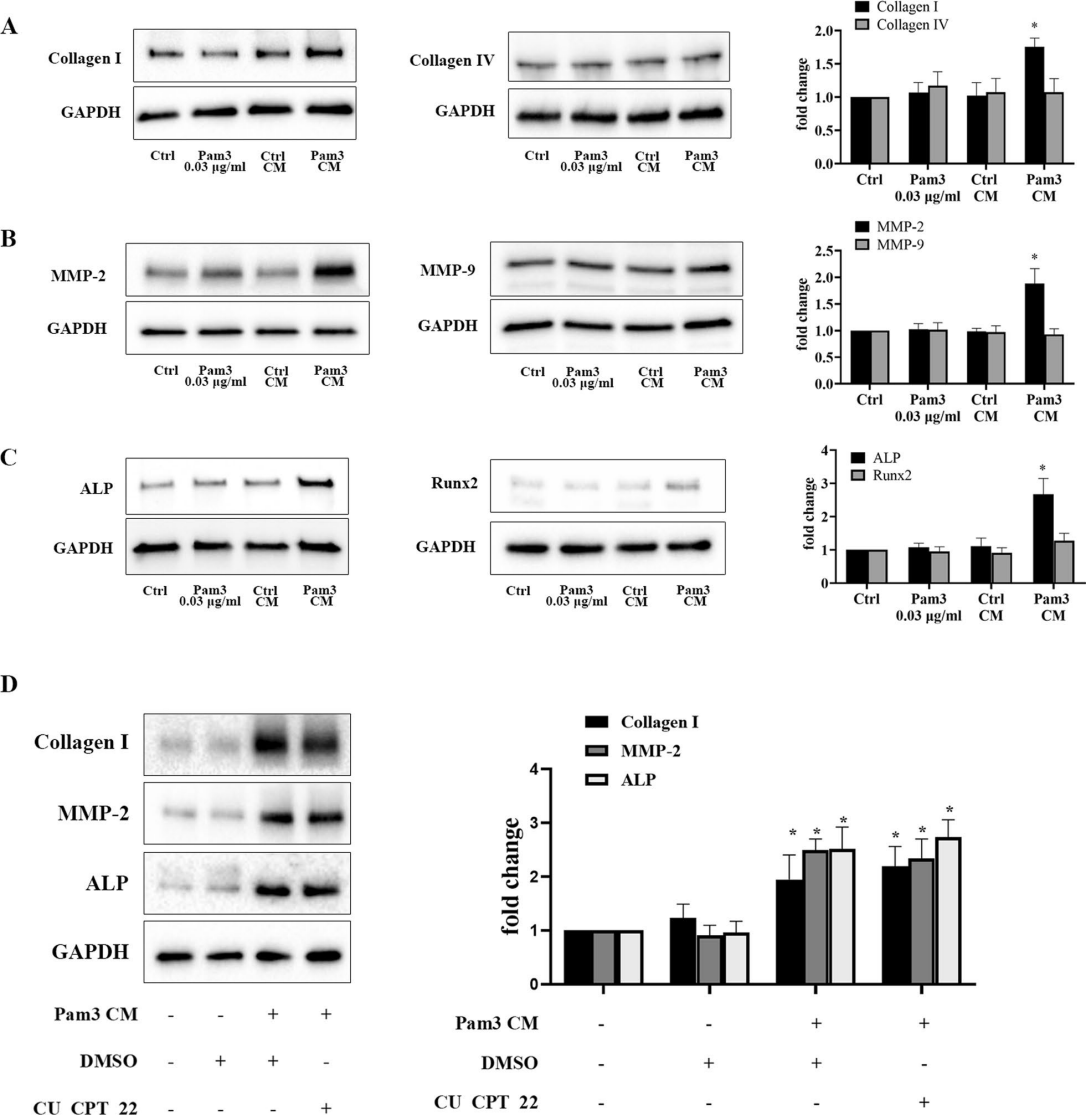
Pam3 CM up-regulates fibrogenic and osteogenic mediators in AVICs independent of TLR2. **A–C** AVICs were exposed to Pam3 (0.03 μg/ml), control CM or Pam3 CM for 48 h. Levels of collagen I, collagen IV, MMP-2, MMP-9, ALP and RUNX2 were determined by immunoblotting. Representative immunoblots and densitometric data show that Pam3 CM selectively up-regulated collagen I, MMP-2 and ALP in AVICs among the fibrogenic and osteogenic factors examined while control CM or a low concentration of Pam3 had no effect. **D** AVICs were pretreated with TLR2 inhibitor CU CPT 22 (10 µM) or DMSO for 1 h and then exposed to Pam3 CM treatment for 48 h. Inhibition of TLR2 in AVICs did not alter the effect of Pam3 CM on the upregulation of collagen I, MMP-2 and ALP. Data are presented as mean ± SEM. n = 5 cell isolates from distinct donor valves in each group. *P < 0.05 vs. untreated control

We treated AVICs Pam3 CM or control CM for 48 h to confirm the effect of Pam3 CM on the fibrogenic and osteogenic responses. Levels of fibrogenic markers, collagen and matrix metalloproteinases (MMP), as well as osteogenic markers, alkaline phosphatase (ALP) and runt-related transcription factor 2 (RUNX2) were examined using immunoblotting. Protein levels of collagen I, MMP-2 and ALP were markedly increased in human AVICs following an exposure to Pam3 CM (Fig. 2A–C). Levels of collagen IV and MMP9 were unchanged. While RUNX2 level was slightly increased by the treatment with Pam3 CM, the change was insignificant. Together, these data suggest that Pam3 CM selectively up-regulates the expression of collagen I, MMP-2 and ALP to enhance the fibrocalcific activity in human AVICs.

To determine whether the low level of TLR2 agonist in Pam3 CM is responsible for inducing the expression of pro-fibrogenic and pro-osteogenic factors, we applied Pam3 (0.03 μg/ml) to treat human AVICs for 48 h. This concentration of TLR2 agonist failed to enhance the expression of collagen I, MMP-2 and ALP (Fig. 2A–C). To determine whether TLR2 is involved in mediating the fibrogenic and osteogenic responses to Pam3 CM, we treated AVICs with TLR2 inhibitor CU CPT 22 prior to exposing AVICs to Pam3 CM. Immunoblotting data revealed that blocking TLR2 had no effect on Pam3 CM-induced expression of collagen I, MMP-2 and ALP in AVICs (Fig. 2D). These results demonstrate that the pro-fibrocalcific effect of Pam3 CM on AVICs is not due to the activation of TLR2. It likely that pro-inflammatory mediators from activated monocytes are responsible for the pro-fibrocalcific effect of Pam3 CM.

### RANTES and TNF-α secreted by activated monocytes mediate the up-regulation of AVIC fibrogenic and osteogenic responses

**Fig. 3:**
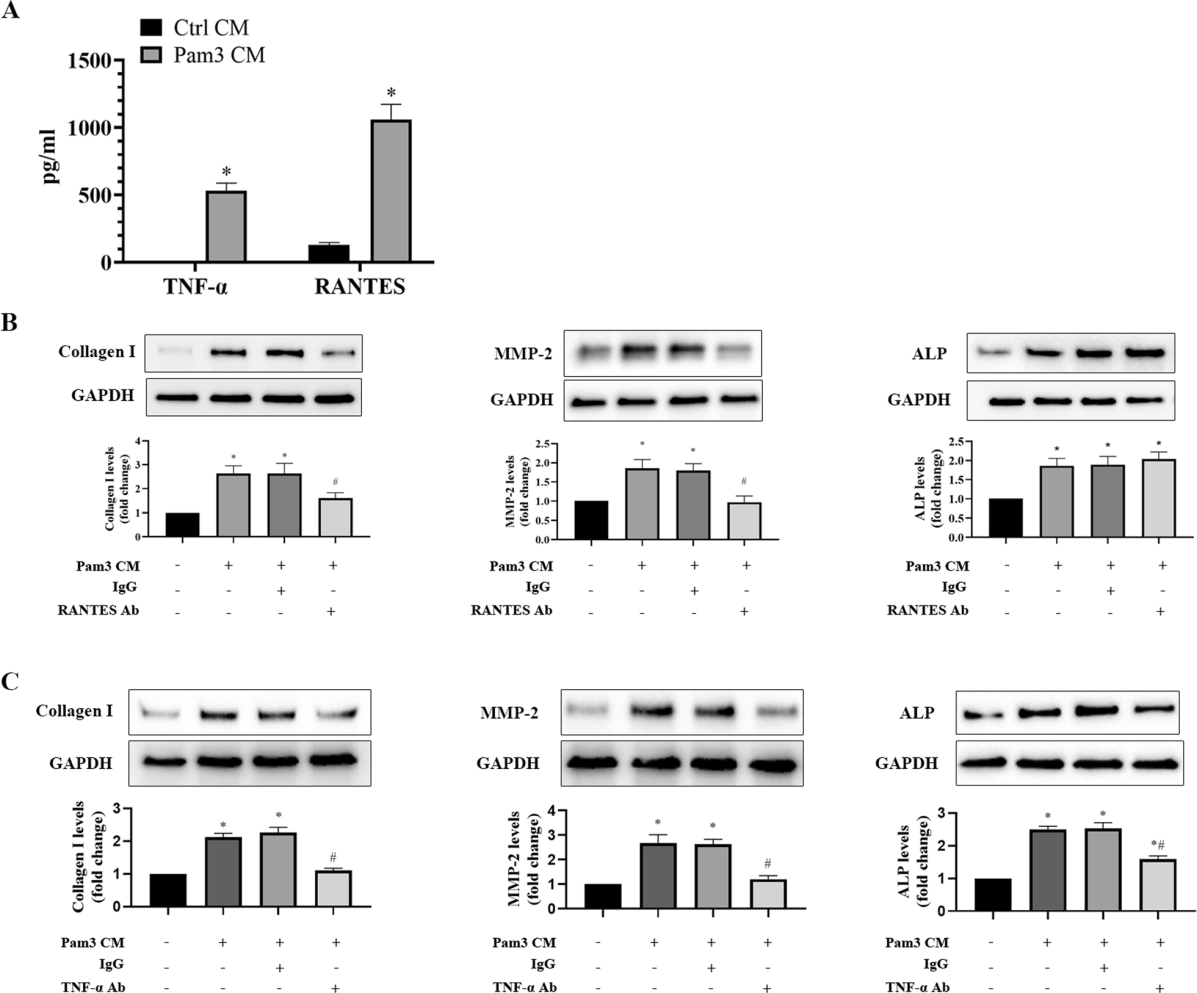
RANTES contributes to the up-regulation of fibrogenic mediators, and TNF-α mediates Pam3 CM-induced up-regulation of both fibrogenic and osteogenic mediators. **A** ELISA assay found that Pam3 CM has greater levels of TNF-α and RANTES in comparison to control CM. Data are presented as mean ± SEM. n = 5, *P < 0.05 vs. control CM. **B** and **C** AVICs were treated for 48 h with Pam3 CM pre-incubated with neutralizing antibodies (10 μg/ml) against RANTES or TNF-α. Non-immune mouse IgG (10 μg/ml) was added to Pam3 CM as a control. Neutralization of RANTES reduced the potency of Pam3 CM on the expression of collagen I and MMP-2 while neutralization of TNF-α attenuated the effect of Pam3 CM on the levels of collagen I, MMP-2 and ALP. Data are presented as mean ± SEM. n = 5 cell isolates from distinct donor valves in each group. *P < 0.05 vs. untreated control. ^*#*^*P* < 0.05 vs. Pam3 CM and Pam3 CM with IgG

Our recent multiplex ELISA analyses revealed that RANTES and TNF-α were markedly higher in Pam3 CM compared to control CM, and these two cytokines displayed greater increase among all pro-inflammatory mediators analyzed (Zhang et al. 2020). In this study, we performed ELISA analyses of the Pam3 CM and confirmed that Pam3 CM have greater levels of RANTES and TNF-α in comparison to control CM (Fig. 3A). We then examined whether these two cytokines play a role in Pam3 CM-induced fibrogenic and osteogenic responses in AVICs. We exposed human AVICs to Pam3 CM pretreated with a neutralizing antibody against RANTES or TNF-α. As shown in Fig. 3B, neutralization of RANTES in Pam3 CM markedly reduced its up-regulation of collagen I and MMP-2 in human AVICs, but had no influence on its effect on ALP level (Fig. 3C). In contrast, neutralization of TNF-α reduced the effect of Pam3 CM on ALP, as well as collagen I and MMP-2 in human AVICs (Fig. 3C). These results demonstrate that both RANTES and TNF-α contribute to the induction of AVIC fibrogenic response by Pam3 CM and that TNF-α mediates the induction of AVIC osteogenic response.

### The JNK pathway mediates AVIC fibrogenic response to RANTES and TNF-α while the NF-κB pathway mediates AVIC osteogenic response to TNF-α

**Fig. 4:**
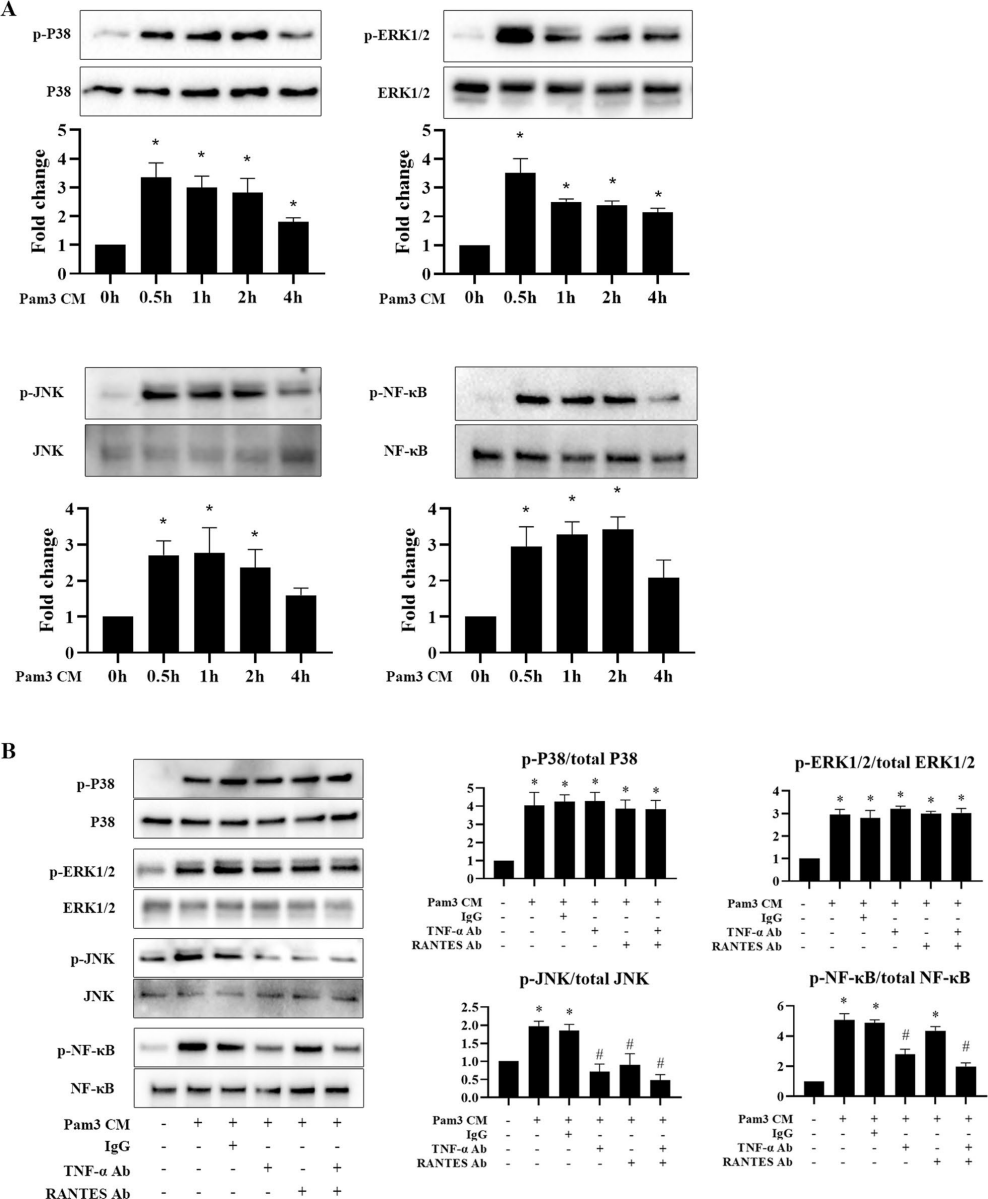
RANTES is involved in Pam3 CM-induced JNK phosphorylation, and TNF-α mediates the effect of Pam3 CM on JNK and NF-κB phosphorylation. **A** AVICs are treated with Pam3 CM for 0.5, 1, 2 or 4 h. Representative immunoblots and densitometric data show that Pam3 CM induces the phosphorylation of p38, ERK1/2, JNK and NF-κB. *P < 0.05 vs. 0 h. Data are presented as mean ± SEM. n = 5 cell isolates from distinct donor valves in each group. **B** AVICs were treated for 1 h with Pam3 CM pre-incubated with neutralizing antibodies (10 μg/ml) against TNF-α, RANTES or both. Neutralization of TNF-α attenuated the effect of Pam3 CM on JNK and NF-κB phosphorylation. Neutralization of RANTES attenuated the effect of Pam3 CM on JNK phosphorylation. *P < 0.05 vs. untreated control. ^*#*^*P* < 0.05 vs. Pam3 CM or Pam3 CM treated with non-immune IgG. Data are presented as mean ± SEM. n = 5 cell isolates from distinct donor valves in each group

We sought to identify the signaling pathways activated by Pam3 CM in human AVICs by examining the activation of NF-κB and three mitogen-activated protein kinase (MAPK) signaling pathways including ERK1/2, Jun N-terminal kinases (JNK) and p38. As shown in Fig. 4A, the phosphorylation of p38, ERK1/2, JNK and NF-κB in AVICs began to increase at 30 min of Pam3 CM treatment and remained elevated in cells exposed to Pam3CM for 2 or 4 h. We then determined whether RANTES and TNF-α in the Pam3 CM are responsible for their activation. The results in Fig. 4B show that neutralization of RANTES in Pam3 CM only suppressed its effect on JNK activation AVICs. In contrast, neutralization of TNF-α in Pam3 CM attenuated its effect on the activation of both JNK and NF-κB in AVICs. Neutralization of either RANTES or TNF-α in Pam3 CM had no effect on its activation of p38 and ERK1/2. It is noteworthy that neutralization of both RANTES and TNF-α in Pam3 CM negated its ability to activate JNK, and neutralization of TNF-α alone in Pam3 CM essentially abolished its induction of NF-κB activation.

**Fig. 5:**
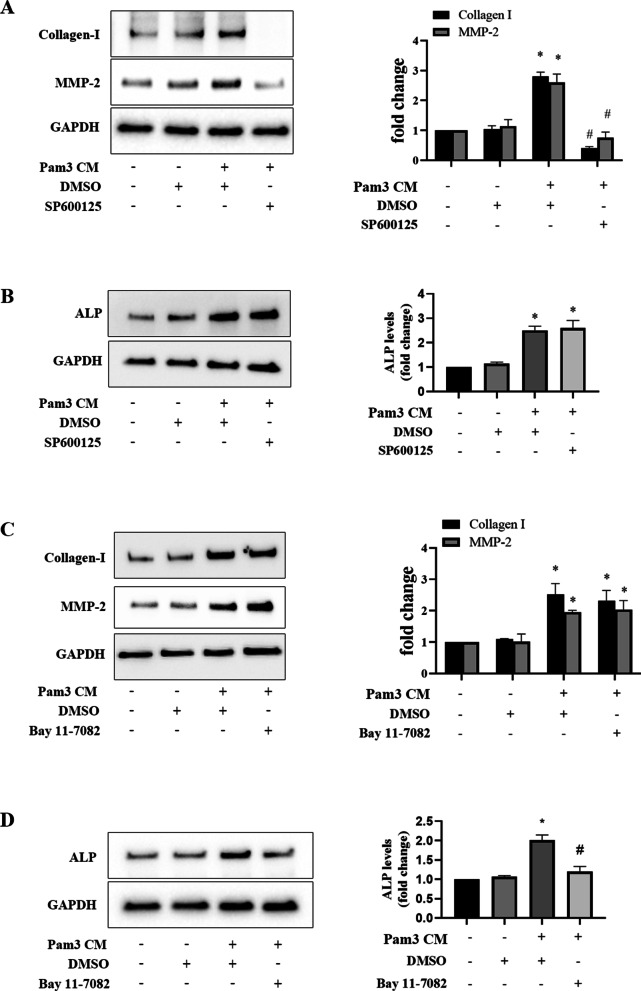
JNK mediates fibrogenic mediator production, while NF-κB mediates the osteogenic mediator production. **A** and **B** AVICs were treated with JNK inhibitor SP600125 (10 μmol/L) or DMSO for 1 h followed by Pam3 CM treatment for 48 h. Inhibition of JNK abolished Pam3 CM-induced collagen I and MMP-2 up-regulation, but had no effect on ALP up-regulation. **C** and **D** AVICs were treated with NF-κB inhibitor Bay 11-7082 (10 μmol/L) or DMSO for 1 h followed by Pam3 CM treatment for 48 h. Inhibition of NF-κB suppressed ALP up-regulation by Pam3 CM, but had no effect on the up-regulation of collagen I and MMP-2 induced. All data are presented as mean ± SEM. n = 5 cell isolates from distinct donor valves in each group. *P < 0.05 vs. untreated control. ^*#*^*P* < 0.05 vs. Pam3 CM with DMSO

To determine the role of JNK and NF-κB in mediating the pro-fibrocalcific effect of Pam3 CM on human AVICs, we applied a JNK inhibitor (SP600125) and a NF-κB inhibitor (Bay 11-7082) to AVICs before being exposed to Pam3 CM. As shown in Fig. 5A and B, inhibition of JNK abolished Pam3 CM-induced production of collagen I and MMP-2, but had no effect on ALP production. Figure 5C and D show that NF-κB inhibitor Bay 11-7082 abrogated Pam3 CM-induced ALP production in AVICs, but it had no effect on the production of collagen I and MMP-2. Taken together, the results show that Pam3 CM induces AVIC fibrogenic response via activation of JNK by RANTES and TNF-α, and enhances AVIC osteogenic response via activation of NF-κB by TNF-α.

## Discussions

CAVD is characterized by progressive thickening of aortic calve leaflets due to fibrosis and calcification (Miller et al. 2011; Weiss et al. 2013) and fibrocalcification will eventually result in valvular dysfunction and heart failure. Currently, the scientific consensus is that chronic valvular inflammation is a critical factor promoting CAVD progression (Cho et al. 2018). In this regard, pro-inflammatory mediators are demonstrated to induce fibrogenic and osteogenic responses in human AVICs (Meng et al. 2008; Song et al. 2012; Yao et al. 2020; Ohukainen et al. 2018). Monocyte/macrophage accumulation occurs in diseased aortic valves (Zhou et al. 2020). Further, activated monocytes have been found to enhance the expression of pro-inflammatory and pro-osteogenic mediators in AVICs (Zhang et al. 2020; Li et al. 2016, 2017b). However, the mechanism underlying the effect of activated monocytes and macrophages on AVIC fibrocalcific activity remains incompletely understood.

The present study demonstrates: (1) Conditioned medium derived from monocytes activated through TLR2 is capable of inducing fibrocalcification in human AVICs. (2) Pro-inflammatory cytokines, RANTES and TNF-α, secreted by activated monocytes up-regulate the production of fibrogenic and osteogenic mediators in human AVICs. (3) RANTES and TNF-α mediate the activation of JNK and NF-κB signaling pathways by the conditioned medium from activated monocytes. (4) JNK activation in AVICs by both RANTES and TNF-α is responsible for the pro-fibrogenic effect of activated monocytes, whereas NF-κB activation by TNF-α is responsible for the pro-osteogenic effect of activated myocytes. The results of this study provide new insights into the mechanism by which activated monocytes induce aortic valve fibrocalcification and highlight the role of monocytes and macrophages in the pathobiology of CAVD.

Infiltrated monocytes and tissue macrophages enhance tissue inflammatory activity in chronic diseases (Suhrbier 2019; Sun et al. 2021). Our recent study found that CM from activated monocytes enhances AVIC inflammatory activity (Zhang et al. 2020). Interestingly, we observed in the present study that CM from TLR2-activated monocytes increases collagen and calcium deposition in human AVICs. Collagen deposition is a biomarker of in vitro fibrogenic activity, and calcium deposition is a biomarker of in vitro osteogenic activity (Singh and Torzewski 2019; Liu and Xu 2016). Therefore, elevated collagen and calcium deposition in AVICs exposed to CM from TLR2-activated monocytes indicates that activated monocytes promote aortic valve fibrocalcification via a paracrine mechanism. In addition to greater collagen deposition, the production of collagen I and MMP-2 is increased, indicating ECM protein remodeling. It appears that up-regulated collagen I production contributes to the increase in collagen deposition since collagen IV levels are essentially unchanged. Associating with the greater calcium deposition, ALP levels are significantly increased. Although the levels of RUNX2 are slightly increased, the change does not reach statistical significance. This differential expression of osteogenic mediators indicates AVIC transition to early osteoblast-like phenotype in response to the CM from TLR2-activated monocytes since ALP is a biomarker of early osteoblast, and RUNX2 is a biomarker of mature osteoblast (Yang et al. 2009; Song et al. 2020).

It should be noted that a low level of TLR2 agonist (Pam3CSK4, 0.03 μg/ml) is present in the CM from TLR2-activated monocytes. We previously observed that Pam3CSK4 at 0.1 μg/ml is capable of inducing ALP expression in human AVICs (Zeng et al. 2017). In evaluation of the effect of low level of Pam3CSK4, we noted that this TLR2 agonist at 0.03 μg/ml is inadequate to up-regulate collagen I and ALP. Thus, it should be the factors secreted by activated monocytes that enhance fibrocalcification in human AVICs. In this study, we confirmed that monocytes exposed to TLR2 agonist release high levels of RANTES and TNF-α. Therefore, we determined the role of RANTES and TNF-α in AVIC expression of fibrogenic and osteogenic mediators.

Interestingly, neutralization of RANTES in the CM selectively suppresses collagen I and MMP-2 expressions in AVICs. Thus, RANTES secreted by activated monocytes plays a critical role in the induction of fibrogenic response in human AVICs. A number of in vivo and in vitro studies have demonstrated that RANTES is pro-fibrogenic, and recombinant RANTES protein is capable of inducing fibrotic changes in different cells and organs (Wang et al. 2021; Berres et al. 2010; Schwabe et al. 2003). Our observation is consistent with previous reports that RANTES modulates ECM protein production and remodeling (Agere et al. 2017; Montecucco et al. 2012; Wintges et al. 2013). The findings of the present study indicate that endogenous RANTES produced by monocytes and macrophages plays a mechanistic role in aortic valve fibrosis associated with CAVD progression. However, it remains unclear from our study what level of RANTES in the valvular microenvironment is sufficient to induce the fibrogenic response in human AVICs.

Moreover, the results of TNF-α neutralization experiments show that TNF-α in the CM is involved in up-regulating the expression of collagen I, MMP-2 and ALP. A previous study found that TNF-α induces the osteogenic response in AVICs (Kaden et al. 2005). Our results demonstrate that TNF-α from activated monocytes is pro-fibrogenic as well. Overall, the results of the present study demonstrate that both RANTES and TNF-α mediate the pro-fibrogenic effect of the CM on human AVICs, and that TNF-α is also pro-osteogenic to human AVICs. Together, these findings indicate that pro-inflammatory cytokines secreted by activated monocytes play a crucial role in enhancing AVIC fibrocalcific activity associated with CAVD progression. Apparently, inflammatory mediators modulate fibrogenic and osteogenic activities in human AVICs. However, it is unclear from the present study and the literature whether an interaction between fibrogenic activity and osteogenic activity exists in human AVICs. Limited evidence suggests that collagen I and MMP-2 may induce ECM remodeling to modulate the osteogenic activity in aortic valve (Weiss et al. 2013; Leopold 2012; Chen and Simmons 2011). Future studies are needed to examine the potential interaction between fibrogenic response and osteogenic response in aortic valve cells.

Both RANTES and TNF-α induce cellular inflammatory responses through the activation of MAPK and NF-κB signaling pathways (Antwerp et al. 1996; Kim et al. 2006), and these signaling pathways have been identified as the primary modulators of several chronic inflammatory diseases (Chen et al. 2018). Previous studies, including ours, have reported that MAPK and NF-κB play important roles in CAVD pathobiology by elevating the fibrogenic and osteogenic responses in human AVICs (Zhan et al. 2015; Munjal et al. 2014). In the present study, we observed that CM from activated monocytes induces the phosphorylation of p38, ERK1/2, JNK and NF-κB in human AVICs. More importantly, JNK phosphorylation was abrogated by neutralization of RANTES or TNF-α, while NF-κB phosphorylation was suppressed only by neutralization of TNF-α. Thus, both RANTES and TNF-α may utilize the JNK pathway to up-regulate AVIC fibrogrnic activity, and TNF-α may also use the NF-κB signaling pathway to up-regulate AVIC osteogenic activity.

Indeed, inhibition of JNK abrogated CM-induced expression of collagen-I and MMP-2, whereas NF-κB inhibitor had no effect on the expression of these two pro-fibrogenic mediators. In contrast, NF-κB inhibitor greatly reduced ALP expression in AVICs exposed to the CM from activated monocytes. This observation suggests a major role of the NF-κB pathway in mediating the pro-osteogenic effect of TNF-α in the CM and supports the concept that NF-κB plays a critical role in modulating valvular osteogenic activity (Meng et al. 2008; Li et al. 2017a; Zhan et al. 2017). It is noteworthy that phosphorylation of p38 and ERK1/2 is also induced by the CM from activated monocytes. Neither of these two MAPKs may have a significant role in mediating the CM effects observed in the present study since inhibition of JNK and NF-κB essentially abrogates the pro-fibrogenic and pro-osteogenic effects of the CM from activated monocytes.

It should be noted that a limitation of the present study is the use of THP-1 cells to evaluate the impact of monocyte-derived inflammatory mediators on the fibrocalcific activity of human AVICs. We elected to use THP-1 monocyte cell line, not monocytes isolated from healthy human donors, in order to eliminate the confounding factor introduced by variable responses of monocytes from different donors. While several studies have compared the TLR-induced inflammatory responses in THP-1 cells and monocytes isolated from healthy donors and found that their responses are comparable (Wu et al. 2017; Sharif et al. 2007), a subtle phenotypic difference may exist between cells from the two sources.

## Conclusions

**Fig. 6:**
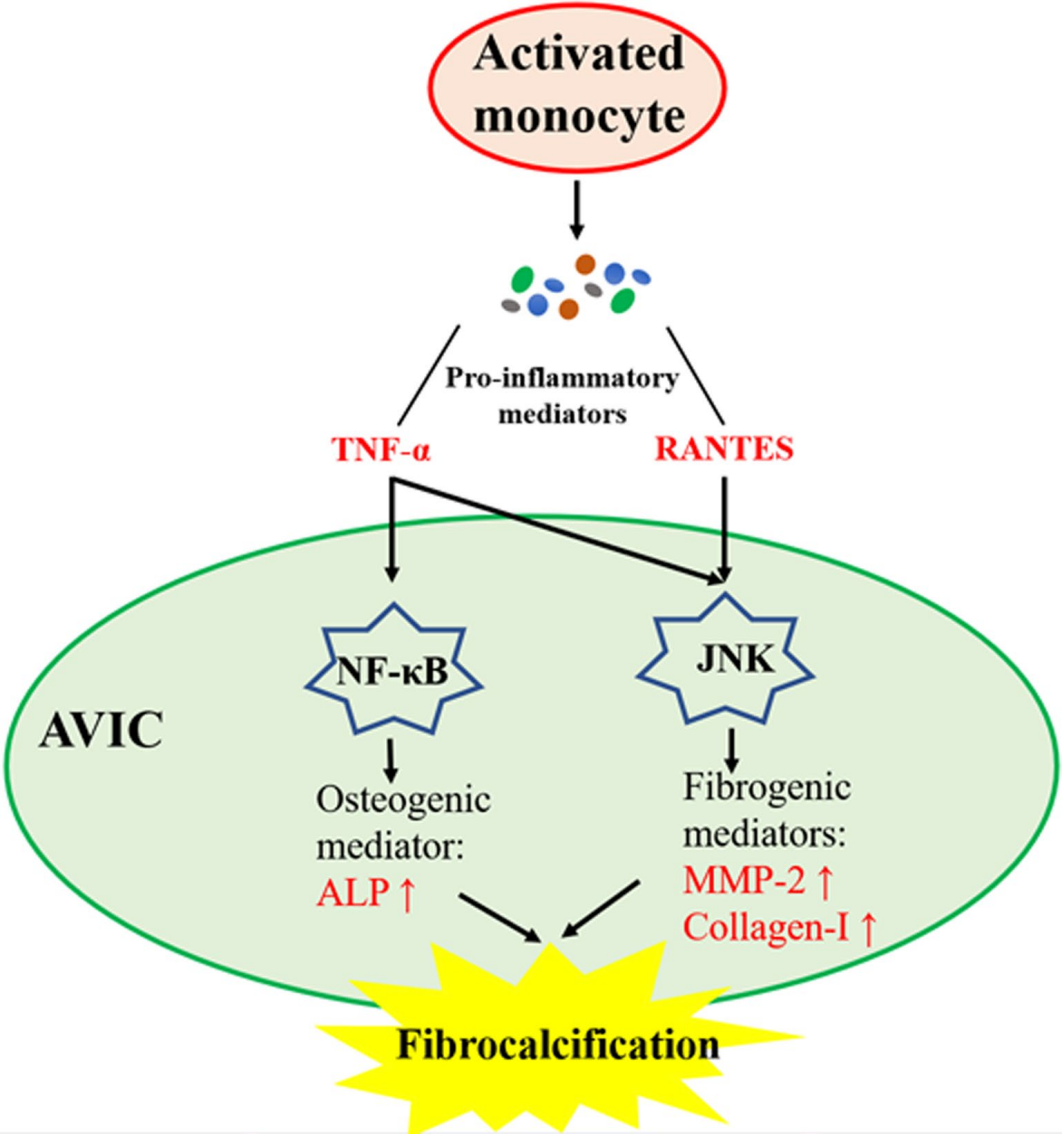
Schematic diagram depicting the mechanism underlying AVIC fibrocalcification induced by medium conditioned by activated monocytes. Pro-inflammatory mediators produced by monocytes activated by TLR-2 agonist induce AVIC fibrocalcification involving up-regulation of fibrogenic and osteogenic mediators. RANTES contributes to the fibrogenic response induced by Pam3 CM through activation of JNK, and TNF-α mediates the pro-fibrogenic and pro-osteogenic effects of Pam3 CM through activation of both JNK and NF-κB

In summary, this study demonstrates that activated monocytes enhance AVIC fibrocalcific activity via a paracrine mechanism. Both RANTES and TNF-α secreted by activated monocytes up-regulate the fibrogenic activity in human AVICs via JNK signaling, and TNF-α from activated monocytes also activates NF-κB to up-regulate the osteogenic activity in human AVICs. These novel findings suggest that activated monocytes may accelerate the progression of CAVD via promotion of aortic valve fibrocalcification (Fig. 6).

## Data Availability

Not applicable.

## Abbreviations

CAVD: Calcific aortic valve disease
AVIC: Aortic valve interstitial cell
TLR: Toll-like receptor
CM: Conditioned medium
Pam3CSK4: Pam3CysSerLys4
Pam3 CM: Pam3CSK4 conditioned medium
Control CM: Control conditioned medium
RANTES: Regulated on activation, normal T cell expressed and secreted
TNF-α: Tumor necrosis factor-α
JNK: C-Jun N-terminal kinase
NF-κB: Nuclear factor kappa-B
MAPK: Mitogen-activated protein kinase
GAPDH: Glyceraldehyde 3-phosphate dehydrogenase
DMSO: Dimethyl sulfoxide
CU CPT 22: 3,4,6-Trihydroxy-2-methoxy-5-oxo-5H-benzocycloheptene-8-carboxylic acid hexyl ester
MMP: Matrix metallopeptidase
ALP: Alkaline phosphatase
Runx: Runt-related transcription factor
ELISA: Enzyme-linked immunosorbent assay
